# Structural differences between unannealed and expanded high-density amorphous ice based on isotope substitution neutron diffraction

**DOI:** 10.1080/00268976.2019.1649487

**Published:** 2019-08-09

**Authors:** Katrin Amann-Winkel, Daniel T. Bowron, Thomas Loerting

**Affiliations:** a Institute of Physical Chemistry, University of Innsbruck, Innsbruck, Austria; b Department of Physics, AlbaNova University Center, Stockholm University, Stockholm, Sweden; c ISIS Facility, Rutherford Appleton Laboratory, Oxon, UK

**Keywords:** Amorphous ice, water, neutron diffraction

## Abstract

We here report isotope substitution neutron diffraction experiments on two variants of high-density amorphous ice (HDA): its unannealed form prepared *via* pressure-induced amorphization of hexagonal ice at 77 K, and its expanded form prepared *via* decompression of very-high density amorphous ice at 140 K. The latter is about 17 K more stable thermally, so that it can be heated beyond its glass-to-liquid transition to the ultraviscous liquid form at ambient pressure. The structural origin for this large thermal difference and the possibility to reach the deeply supercooled liquid state has not yet been understood. Here we reveal that the origin for this difference is found in the intermediate range structure, beyond about 3.6 Å. The hydration shell markedly differs at about 6 Å. The local order, by contrast, including the first as well as the interstitial space between first and second shell is very similar for both. ‘eHDA’ that is decompressed to 0.20 GPa instead of 0.07 GPa is here revealed to be rather far away from well-relaxed eHDA. Instead it turns out to be roughly halfway between VHDA and eHDA – stressing the importance for decompressing VHDA to at least 0.10 GPa to make an eHDA sample of good quality.

## GRAPHICAL ABSTRACT

**Figure:**
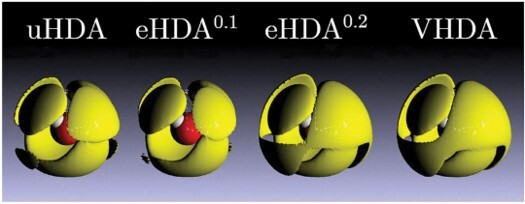

## Introduction

High-density amorphous ice (HDA) was discovered by Mishima *et al.* in the 1980s [1]. They coined the concept of polyamorphism by demonstrating that HDA represents a second form of amorphous ice, distinct from low-density amorphous ice (LDA) that has long been known [2]. Soon thereafter the first X-ray [3] and neutron scattering studies related to HDA were published [4]. The study of the site-site radial distribution functions (RDFs) in amorphous ices has traditionally been done using the technique of isotope-substitution neutron diffraction. These studies were pioneered in collaboration with Alan Soper in the early 2000s. Back then RDFs for LDA and HDA were first reported based on data recorded at 80 K at the SANDALS instrument at the ISIS Neutron and Muon Source for research at the Rutherford Appleton Laboratory [5]. At about the same time the third distinct form of amorphous ice, very-high density amorphous ice (VHDA), was discovered [6]. Its microscopic structure was again reported in collaboration with Alan Soper [7]. These three amorphous ices all share the same basic building block – the Walrafen pentamer. This simply is a central water molecule tetrahedrally surrounded by four neighbouring water molecules directly linked to the central molecule through hydrogen bonds. The distinction between LDA, HDA and VHDA is found in the interstitial space between the first and second hydration shell around the central water molecule. While this is empty in case of LDA [5], one water molecule has moved from the second shell to an interstitial site in case of HDA [5] and two molecules in case of VHDA [7]. There is no direct hydrogen-bond from the interstitial water molecules to the central water molecule, and so the coordination number can be described as 4 + 0, 4 + 1 and 4 + 2 for LDA, HDA and VHDA, respectively. Please note that each interstitial water molecule is, of course, also tetrahedrally surrounded, just like every single water molecule in amorphous ices.

In other words, the site-site RDFs deduced from isotope substitution neutron diffraction at ambient pressure clearly reveal LDA, HDA and VHDA to be structurally distinct. The most characteristic difference is revealed in the OO pair distribution function *g*_OO_(*r*), where the second peak shifts from ∼4.5 Å in LDA to ∼3.8 Å in HDA and ∼3.4 Å in VHDA [7]. By contrast, the same technique does not reveal any structural difference between hyperquenched glassy water (HGW), annealed amorphous solid water (ASW) and LDA [8]. All site-site RDFs are identical within experimental error. This comes as a surprise since these three amorphous ices are prepared from different starting materials: ASW is deposited from water-vapor [9], HGW is produced from liquid droplets that are cooled at high rates (10^6^–10^7^ K/s) [10] and LDA is produced starting from hexagonal ice *via* HDA [2]. That is, all of these three forms of ice belong to the same family of low-density amorphous ices, whereas HDA and VHDA can be considered families of their own. Consequently, the neutron diffraction studies have revealed three families of amorphous ices, which are known as the three polyamorphic forms of water [11].

The family of low-density amorphous ices was investigated in more detail in 2009, again by isotope substitution neutron diffraction [12]. While this study confirms that low-density amorphous ices belong to the same family, some small structural differences between two preparations of LDA could be discovered. There are no differences in the first two hydration shells, showing that both preparations belong to the LDA family. However, at the intermediate length scale, beyond the second shell differences were found in *g*_OO_(*r*). These are most pronounced approximately at a distance of about 10–15 Å from the central molecule. One form appears to be slightly more ordered, with sharper peaks for the third and subsequent hydration shells. This may be due to this form being more relaxed than the other LDA preparation [12].

Around this time it has also become clear that several substates exist for HDA. Nelmes *et al*. were able to show that relaxation takes place upon heating HDA at ‘low’ pressures near 0.2 GPa [13]. This relaxation causes a slight expansion of HDA, so that Nelmes *et al*. introduced the distinction between unannelaed HDA (uHDA), as originally prepared by Mishima *et al.* [1], and expanded HDA (eHDA) [13]. They showed that eHDA is slightly expanded compared to uHDA based on the shift of the first diffraction maximum to a higher d-spacing. Following the transition to LDA at ambient pressure they showed an increase in thermal stability, dependent on the preparation pressure. Mishima showed previously that the thermal stability of HDA increases by direct amorphization at high temperature or heating HDA samples at high pressure up to 150 K [14]. However, these samples show a 9% higher density than HDA and, therefore, belong to the family of VHDA ices [6]. The densities of uHDA and eHDA instead are similar, specifically 1.15 g/cm^3^ for uHDA and 1.13 g/cm^3^ for eHDA as measured by flotation in a cryo-mixture of liquid argon and nitrogen [15]. The two HDA states further show similar X-ray [16,17] and neutron diffraction patterns [11,18]. The drastically enhanced thermal stability of eHDA compared to uHDA is surprising and remarkable in view of the structural similarity.

Winkel *et al*. then made eHDA on a decompression pathway starting from VHDA at 140 K [18]. They showed that VHDA converts to eHDA in the pressure range from about 0.3 to 0.07 GPa [18]. Studying the transition to LDA at ambient pressure with differential scanning calorimetry (DSC) they found an increase in thermal stability with decreasing recovery pressure. eHDA shows the highest thermal stability and transforms under pressure directly to LDA [13] *via* a first order transition involving sharp interfaces between two amorphous ices [19,20] as well as nucleation and growth of one amorphous ice in the matrix of the other [21,22].

eHDA soon thereafter gained importance. By contrast to uHDA it can be heated beyond its glass-to-liquid transition temperature without conversion to LDA even at ambient pressure. A complimentary study of DSC and dielectric measurements determined the glass transition temperature to be at 116 K [23]. That HDA shows diffusive dynamics above ∼116 K was shown using small-angle X-ray scattering and speckle dynamics measurements [21] as well as from isotope substitution and doping experiments [24]. Thus, the high-density liquid can be accessed and studied for long times at around ∼116 K even at ambient pressure. In case of uHDA transformation to the deeply supercooled liquid does not occur since the polyamorphic transition to LDA preempts it. How the glass-transition temperature of HDA changes with pressure was reported by Loerting *et al*. [25]. The liquid nature of HDA above its glass transition temperature under pressures up to 0.3 GPa was recently proven by Stern *et al*. [26]. Andersson *et al*. showed the glass transition of HDA at elevated pressure (1 GPa) to be at 140 K [27]. All these experimental data taken together give good evidence that the ‘isothermal high- to low-density transition at 140 K takes place in the domain of ultraviscous water’ [20]. These claims are contested, though, based on the suggestion that translational motion might not be involved above *T_g_* but rather reorientation [28].

In the following, we discuss the structural differences of two states of high-density amorphous ice (HDA), namely unannealed HDA (uHDA) and expanded HDA (eHDA). The latter is the well relaxed form and limiting structure of the high density state. uHDA is the amorphous ice that is produced directly by amorphization of hexagonal ice at liquid nitrogen temperature. The uHDA state is not annealed, i.e. has never reached higher temperatures than 80 K. For comparison we also include data for VHDA, which is the parent material from which HDA emerges, and a decompressed eHDA sample that is decompressed only to 0.20 GPa, but not 0.07 GPa. Decompression to 0.07 GPa yields the most relaxed eHDA state that then directly converts to LDA in a first-order like transition. It is unclear how well relaxed eHDA states are that are decompressed to higher pressures, and so we investigate also this question based on isotope substitution neutron diffraction. Some of the data presented here are also published in the Ph.D. thesis by Katrin Winkel [29].

## Methods

### Sample preparation

For optimal structure refinement of the neutron scattering data, H/D isotopic substitution is necessary. Therefore, a set of three samples has to be prepared, one sample of pure H_2_O, one of D_2_O (Sigma-Aldrich, 99.9%), and one sample from a 50:50 mixture of H_2_O and D_2_O. The HDA-samples were prepared in a piston cylinder apparatus (ZWICK, model BZ100/TL3S). The uHDA sample was prepared by compression of ice *I*_h_ at 77 K to a pressure of 1.6 GPa, in accordance with the protocol by Mishima *et al*. [30]. The cylinder was prelined with indium foil to avoid pressure drops during the initial compression cycle of HDA formation [1]. The mould was cooled subsequently by pouring liquid nitrogen into the containing vessel and the sample was recovered at 77 K and ambient pressure. The corresponding isotope substitution neutron data are reported in our earlier work and taken from there [8].

**Figure 1.**
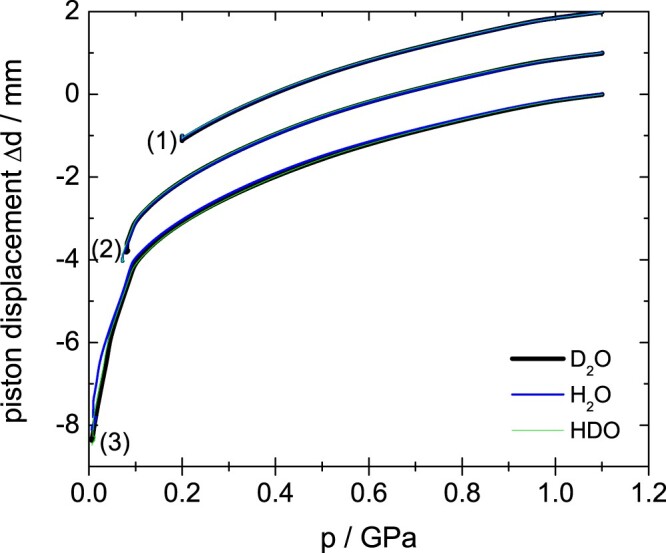
Piston displacement curves for three distinct samples of about 1500 mg, namely (1) eHDA(0.20 GPa), (2) eHDA(0.07 GPa) and (3) LDA. An offset of 1 mm is applied at 1.1 GPa for clarity. Different colours correspond to different isotopologues, as indicated.

eHDA samples were produced as outlined in our earlier work [18,31]. In short, uHDA as described above was heated at 1.1 GPa to 160 K, producing the intermediate state VHDA [6]. The H_2_O sample is then cooled to 140 K and decompressed to 0.07 GPa and quench recovered to ambient pressure, yielding eHDA(0.07 GPa) [18]. Due to the isotope effects, the preparation conditions have to be adapted slightly to reach the same final HDA-state. This has already been discussed in detail in Ref. [31] for D_2_O. Accordingly, the D_2_O-sample was decompressed at 143 K to 0.08 GPa and the HDO-sample at 141.5 K to 0.07 GPa. Figure 1 (set of curves labelled (2)) shows the piston displacement upon decompressing the sample, which is a measure for the volume (or density) of the sample. It is clearly seen that the piston displacement curves for all three isotopologues are on top of each other, demonstrating that the same transition is experienced for H_2_O, D_2_O and HDO samples. Additional samples were prepared decompressing VHDA only to 0.20 GPa (set of curves labelled (1) in Figure 1) and to 0.01 GPa (set of curves labelled (3) in Figure 1). The former produces eHDA(0.20 GPa), which represents an eHDA sample that has not yet reached its full state of relaxation. The density of this eHDA(0.2 GPa) sample is in between eHDA and VHDA and was determined to be 1.20 g/cm^−3^ [15]. The latter corresponds to LDA, where the polyamorphic transition from eHDA to LDA is observed as the sharp piston displacement change (corresponding to density jump) near 0.06 GPa in Figure 1.

**Figure 2.**
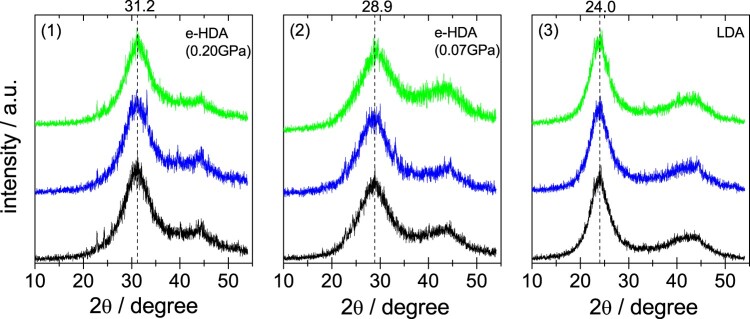
Powder X-ray diffractograms recorded *ex situ* at ∼80 K in vacuum using a Siemens D5000 instrument equipped with a Göbel-mirror for parallel optics and an Anton Paar TTK450 chamber for horizontal sample geometry. Cu-K_α_ was used for (1) eHDA(0.20 GPa), (2) eHDA(0.07 GPa) and (3) LDA. The location of the first halo peak is indicated by a dashed vertical line. Top, middle and bottom (green, blue and black) diffractograms are for HDO, D_2_O and H_2_O, respectively.

The samples were checked for their quality using powder X-ray diffraction at ∼80 K following their production. All measurements reported in Figure 2 are performed *ex situ* on quench-recovered and powdered samples. Two findings are immediately evident upon inspecting the Cu-K_α_ diffractograms: (i) there is no evidence for sharp Bragg-peaks, confirming the X-ray amorphous nature of all samples. The only exception are some very weak Bragg peaks near 22, 24, 26 and 40° – these indicate that a tiny bit of hexagonal ice has condensed upon transfer of the sample from the high-pressure cylinder to the vacuum chamber. (ii) The position of the halo maximum, as indicated by the vertical, dashed lines, is identical for HDO (top green curves), H_2_O (middle blue curves) and D_2_O (bottom black curves). The diffraction angle of 31.2° for the halo maximum in eHDA(0.20 GPa) confirms the above-mentioned nature between VHDA and eHDA(0.07 GPa). For VHDA the halo maximum is about 32.5° (*d *= 2.75 Å) [6], whereas it is around 28.3° (*d *= 3.15 Å) for eHDA in its most relaxed state just prior to the transformation to LDA [18]. After transformation to LDA it is 24.0° (*d *= 3.70 Å). It is important to note, that the first X-ray diffraction maximum of eHDA shows slight variations of ±0.2° due to the challenge to keep the temperature constant to better than ±0.2 K for the decompression experiment, friction in the piston cylinder and ambiguities in reading the exact location of the maximum in Figure 2 due to the noise of the signal. The samples used here for neutron diffraction have to be prepared using a larger sample volume of 1.5 ml, instead of 300 µl in Winkel *et al*. [18], where the larger sample volume might cause friction effects such as pressure gradients during the decompression process. On average, the first halo maximum of eHDA is at 2Θ = 29.3° (*d* = 3.05 Å ± 0.1 Å).

**Figure 3.**
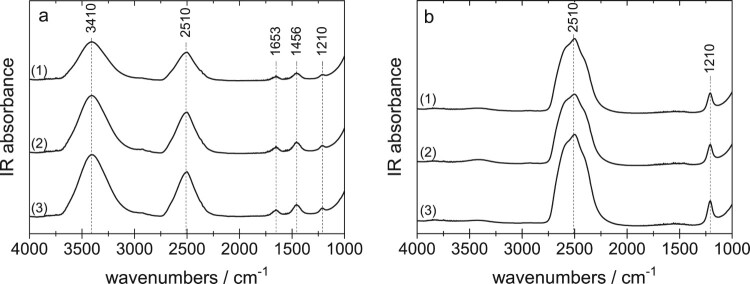
Mid-IR spectra recorded from the liquid samples at room temperature, after measurement at SANDALS and melting sample for (a) HDO and (b) D_2_O. (1) eHDA(0.20 GPa), (2) eHDA(0.07 GPa) and (3) LDA.

The isotopic composition of the samples was determined after the neutron measurement, i.e. after the sample shipped from Austria to UK, and back. All samples were melted, and the liquid was placed between two optical windows and measured at ambient temperature using mid IR spectroscopy (using a Varian Excalibur spectrometer at a resolution of 4 cm^−1^). The spectra for the HDO and D_2_O samples are depicted in Figure 3. The most prominent feature is the broad OD-stretching band centred at 2510 cm^−1^ and the OH-stretching band centred at 3410 cm^−1^. In the pure H_2_O spectra (not shown) there is no band at 2510 cm^−1^, so that it is composed of 100% H_2_O. This is obvious since there is no reason why there should be a D_2_O contamination in H_2_O. For D_2_O samples, however, contamination cannot be excluded because D_2_O might take up H_2_O from humid air. As evident in Figure 3(b) there is a very weak band near 3410 cm^−1^. From a calibration and quantitative analysis of peak areas we deduce the D_2_O sample to be >99.5% pure. The returned HDO samples were determined to have a composition of 49.2% D for both eHDA samples and 48.5% D for the LDA sample, respectively.

Calorimetry measurements comparing such uHDA and eHDA samples were reported in Refs. [11] and [20], respectively. eHDA transforms to LDA at ∼134 K (first exotherm), which is ∼17 K higher than uHDA, see Figure 9 in Ref. [32]. Crystallization takes place at 160 K (second exotherm) for both samples. The structural origin of why eHDA is thermally so much more stable than uHDA is elucidated here.

### Neutron scattering

The neutron scattering experiments with H/D isotopic substitution were performed at the ISIS spallation neutron source (Chilton, Didcot, UK) and follow the protocol established in earlier neutron scattering studies on amorphous ices [8]. The scattering data were collected on the SANDALS Diffractometer [33] and reduced to the interference differential scattering cross section F(Q) using the GudrunN routines [34]. These routines perform the essential background, container scattering, multiple scattering, attenuation and inelastic scattering corrections, and finally normalise the data to the scattering from a known vanadium calibration standard. For measurement, the amorphous ice samples were, under liquid nitrogen, pestled to a powder and loaded into parallel sided TiZr cells for data collection at 80 K and ambient pressure. As the samples were formed from powdered material, the data were also corrected for powder packing fraction as described in Ref. [8]. Data were analysed in the *Q*-range of 0.5–30 Å^−1^. The subsequent structural modelling of the data was performed using Empirical Potential Structure Refinement (EPSR) [35]. The whole procedure is described in detail by Bowron *et al*. [8]. In brief, *F*(*Q*) can be written as

$$F_{N}(Q)=\sum_{\alpha }\sum_{\beta \geq \alpha }\left(2-\delta_{\alpha \beta })c_{\alpha }c_{\beta }\langle b_{\alpha }\rangle \langle b_{\beta }\rangle [S_{\alpha \beta }(Q)-1\right]$$

with *S_αβ_*(*Q*) being the site-site partial structure factors (Faber-Ziman) between atoms of type *α* and *β*, where the coefficients *c_α_*/*c_β_* and *b_α_*/*b_β_* represent concentrations and scattering lengths for the atom types, respectively. To avoid double counting of the like terms within the summation, *δ_αβ_* is the Kronecker delta function. For the case of pure water, the combination of data from three isotopically substituted samples makes it possible for us to extract the three partial structure factors that fully define the atomic pair correlations in the system, *S*_HH_(*Q*), *S*_OH_(*Q*) and *S*_OO_(*Q*) [36]. In all samples studied, the measured neutron diffraction patterns show no sign of Bragg peaks that would have indicated the presence of crystalline material, in accordance with the X-ray data shown in Figure 2.

## Results

**Figure 4.**
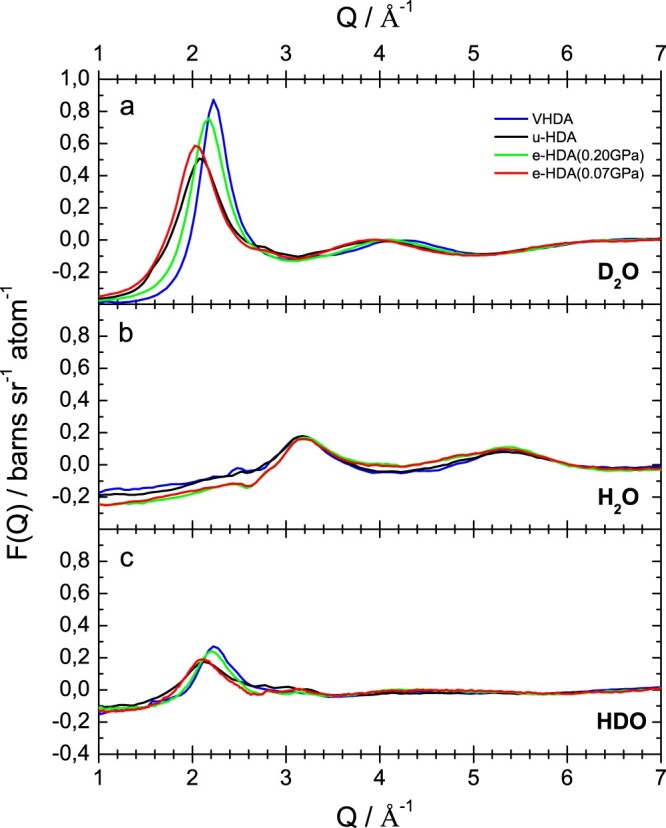
Fully corrected interference differential scattering cross section data, *F*(*Q*), for the samples as indicated.

Figure 4 shows the fully corrected interference differential scattering cross section data, *F*(*Q*), for VHDA (blue lines), eHDA(0.20 GPa) (green lines), eHDA(0.07 GPa) (red lines) and uHDA (black lines). The data are plotted in the Q-range of 1–7 Å^−1^, to show the behaviour of the first diffraction peak at ≈2Å^−1^ (for the (a) D_2_O and (c) HDO case). This prominent feature in the scattering pattern is known to correlate with intermediate range structural organisation in liquids and glasses [8]. Figure 4(a) shows the total static structure factor of the D_2_O-samples. The first diffraction peak has its maximum at *Q* = 2.23 Å^−1^ for VHDA and shifts to *Q* = 2.16 Å^−1^ for eHDA(0.20 GPa) and 2.04 Å^−1^ for eHDA(0.07 GPa). Clearly, eHDA(0.20 GPa) is not even midway through the transition to fully relaxed eHDA(0.07 GPa). The position for uHDA is at 2.1 Å^−1^ for comparison. This small shift to a lower *Q*-value corresponds indirectly to the lower density of eHDA compared to uHDA. Our flotation measurements showed the density of uHDA to be at 1.15 g/cm^3^ and the density of eHDA(0.07 GPa) at 1.13 g/cm^3^ [15]. The differences of these two HDA states are localised in the *Q*-range of the first diffraction peak (*Q* < 2.5 Å^−1^). The first diffraction peak in reciprocal space in *F*(*Q*) of the D_2_O samples, however, contains Fourier components from all molecular distances in real space, but due to its position at relatively low-*Q*, tends to be most sensitive to those correlations in the intermediate distance range from 3 to 6 Å.

Figure 4(b) shows the interference differential scattering cross section, *F*(*Q*), for the H_2_O samples. In the H_2_O-case the first diffraction maximum is located at 3.18 Å^−1^ for all four samples. There is no significant difference within the experimental error between these curves. This effect is due to the negative scattering length of hydrogen *b_H_* as opposed to the positive scattering length of deuterium *b_D_* [37]. As shown above, the interference differential scattering cross-sections *F*(*Q*) is a sum of the three partial structure factors (in case of water *S*_OO_, *S*_OH_ and *S*_HH_) weighted by the respective concentration *c_ab_* and the scattering length *b_ab_* of each atom type. All three partial structure factors have a first diffraction peak at ∼2 Å^−1^ [8]. In D_2_O (Figure 4(a)) both *b_O_* and *b_D_* are positive numbers, so all three terms in *F*(*Q*) are positive, and the first peak in the total structure factor for D_2_O is visible. In H_2_O (Figure 4(b)) *b_O_* is positive but *b_H_* is negative, which means that the first two terms of the sum in *F*(*Q*) are positive, but the third term is negative. The summation is such that in the H_2_O sample the first diffraction peak at ∼2 Å^−1^ disappears in the total structure factor [8]. In the HDO-samples (Figure 4(c)) the first diffraction peak at ∼2 Å^−1^ is visible again. Due to the negative scattering length of hydrogen, as described above, the total scattering intensity in the HDO-samples is smaller compared to the D_2_O-samples. For HDO it actually seems that eHDA(0.20 GPa) is very similar to VHDA, but different from eHDA(0.07 GPa), which is itself similar to uHDA.

**Figure 5.**
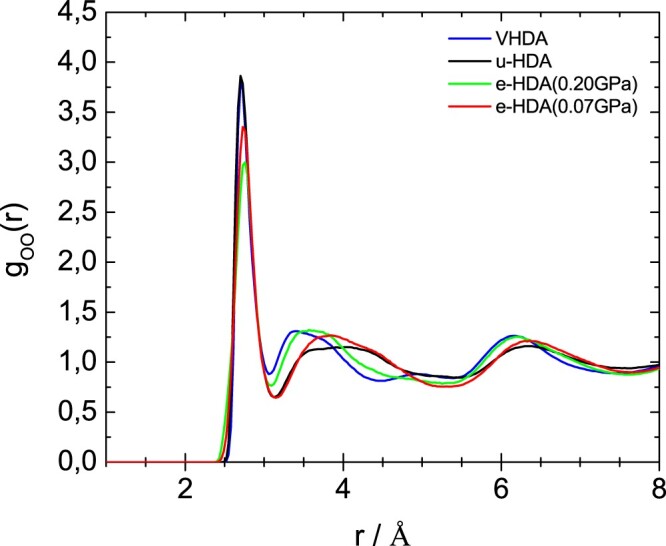
OO partial radial distribution function for the samples as indicated. Please note that some of these curves were published in advance in a review article, to which we contributed Ref. [38].

**Figure 6.**
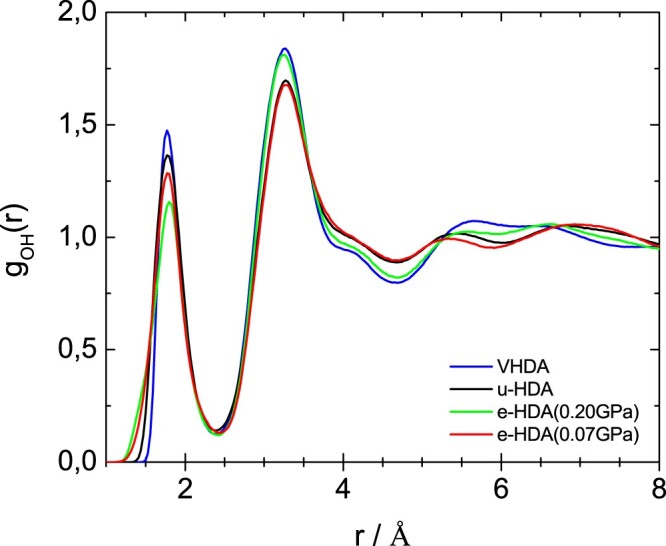
OH partial radial distribution function for the samples as indicated. Please note that some of these curves were published in advance in a review article, to which we contributed Ref. [38].

**Figure 7.**
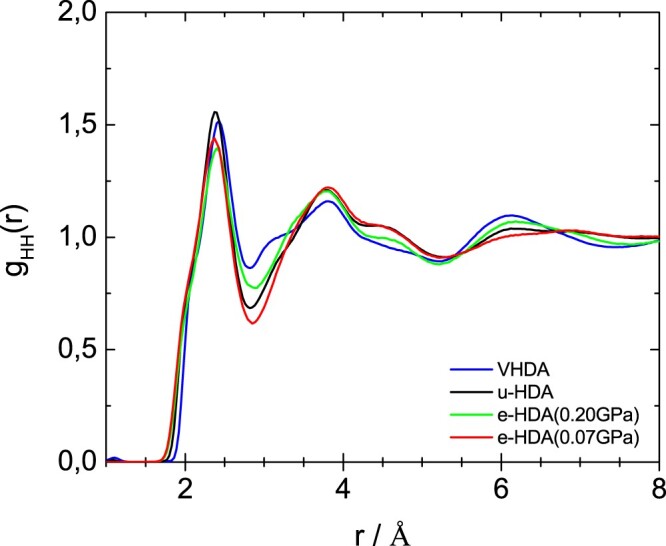
HH partial radial distribution function for the samples as indicated.

Figures 5–7 show the partial radial distribution functions *g*_OO_(*r*), *g*_OH_(*r*) and *g*_HH_(*r*) for the four different samples. In Figure 5 the main difference in OO partial radial distribution function *g*_OO_(*r*) appears for the second peak, which is shifted to larger distance for eHDA(0.07 GPa) and uHDA compared to VHDA and eHDA(0.20 GPa). It is again evident that eHDA(0.20 GPa) is closer to VHDA than it is to eHDA(0.07 GPa). The same trend is also obvious for the third peak near 6 Å. A similar comparison was also undertaken based on high energy X-ray experiments [16,17], showing the same shift between the different amorphous ices and very similar *g*_OO_(*r*) compared to the here presented neutron diffraction data (see SI of ref. 16). OH and HH correlations, however, can only be determined using neutron scattering experiments. In terms of *g*_OH_(*r*), as shown in Figure 6, all four samples are quite similar up to 5 Å, but start to deviate from each other at larger distances. For example, eHDA(0.07 GPa) shows peaks at 5.3 and 7.1 Å, which are shifted in VHDA to 5.5 and 6.6 Å, respectively. That is, the separation between these two peaks is much larger in eHDA(0.07 GPa) than in VHDA. The difference between uHDA and eHDA(0.07 GPa), on the other hand is much smaller. Regarding *g*_HH_(*r*) in Figure 7 the shoulder for VHDA at 3.1 Å is striking, that is absent for the other three samples. Also the peak at ∼6 Å is pronounced in VHDA, but broad and smeared out for eHDA(0.07 GPa). All these features show that the structures progressively change from VHDA to eHDA(0.20 GPa) to eHDA(0.07 GPa). eHDA(0.20 GPa) is roughly halfway between the two. This is in agreement with the calorimetric data, where transformation to LDA was observed to be at 130.5 K for eHDA(0.20 GPa), which is roughly halfway between 126 K for VHDA and 134 K for eHDA(0.07 GPa) [20].

**Figure 8.**
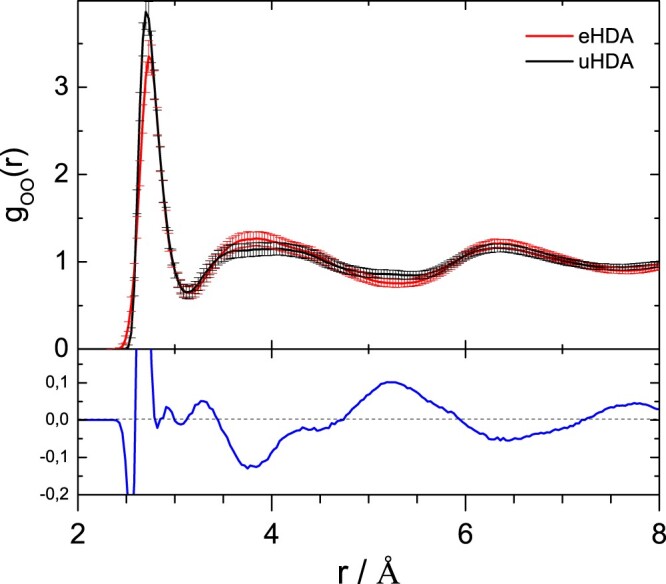
(top) OO partial radial distribution function for uHDA and eHDA(0.07 GPa) to 8 Å, the error bars included represent the ensemble of structural configurations generated in the structure refinement process that are consistent with the supplied diffraction data. (bottom) difference function (*g*_oo_(*r*)_uHDA – *g*_oo_(*r*)_eHDA) (blue line).

By contrast, uHDA and eHDA(0.07 GPa) are more similar to each other. However, they are not identical. And the difference between the two is a necessary condition to rationalise why uHDA transforms to LDA at a much lower temperature, namely at 117 K. To enhance the differences in the OO-coordination Figure 8 shows the error bars derived from the ensemble of configurations in each structure refinement model that are consistent with the supplied diffraction data. The difference function between the two functions highlights the radial dependence of the structural variations between the two systems and this is shown in the lower panel. In this difference function the first peak near 3 Å indicates differences in the nearest oxygen neighbour distances. There are small differences in the length of the hydrogen-bond between uHDA and eHDA(0.07 GPa). However, neutron diffraction is not particularly sensitive to these differences so that this issue needs to be tackled with other techniques such as Raman spectroscopy, which has proven in the past to provide a reliable measure for OO-distances based on the decoupled OH-stretching vibration [6]. This distance was found to be 2.85 Å for VHDA and 2.82 Å for uHDA based on Raman measurements. Further the OO-distances were determined using the first X-ray diffraction peak to be 2.78 Å for eHDA and 2.80 for VHDA [16]. Although the absolute values ascertained by the two methods differ, the general trend of an increasing OO-distance with increasing density between u/eHDA and VHDA is consistent [16]. This is not reflected here in the neutron scattering data. Nevertheless, the difference in the second coordination shell, as seen in Figure 8 around 4 Å, agrees well with the X-ray scattering data [17]. The radial distribution function of eHDA (red line) is enhanced around 3.6 Å, while uHDA (black line) shows a stronger contribution at values between 4 and 5.2 Å, indicating that uHDA contains an increased amount of tetrahedrality compared to eHDA [17]. Further peaks in the difference (blue line) in Figure 8 appear near 6.5 Å (third coordination shell) and 7.7 Å.

**Figure 9.**
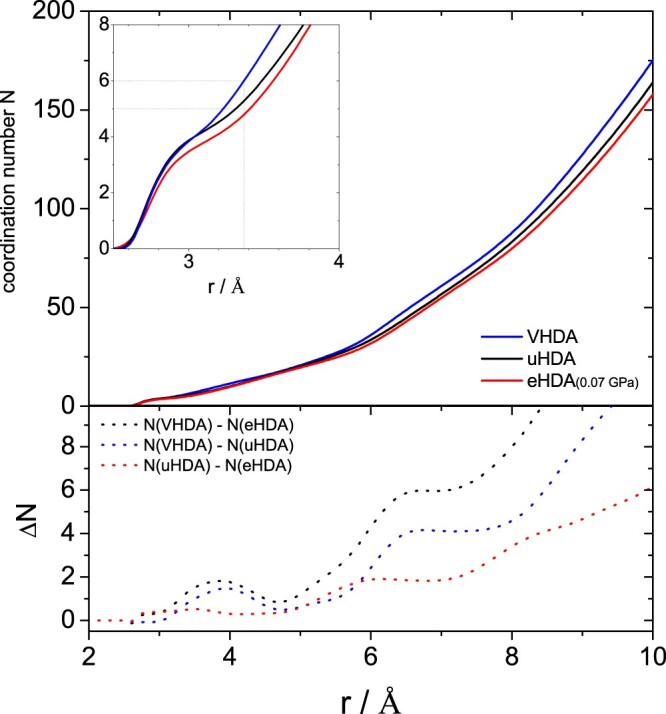
(top) Coordination number as obtained from integration of the OO partial radial distribution function to the given radius r for uHDA (black), VHDA (blue) and eHDA(0.07 GPa) (red). (bottom) differences in coordination number as indicated and calculated from the top curves.

Figure 9 shows the coordination number *N*(*r*) obtained by integration of the partial distribution function *g*_OO_(*r*). The integration provides the average number of atoms within a given distance from the central O-atom [8]. The difference of the running coordination number between uHDA and eHDA(0.07 GPa) is plotted at the bottom (dotted red line). Apart from a small feature near 3 Å the difference is smaller than 1 up to about 5 Å and then rises strongly beyond 5 Å. At about 6 Å the difference reaches a maximum of about 2. That is, the difference between the two is mainly located beyond the second shell, at the intermediate length scale. For comparison, the difference to VHDA (dotted black and blue lines) can be found near 3.7 Å, i.e. in the interstitial space between the first two hydration shells.

## Conclusions

We here compare isotope substitution neutron diffraction data for several sub-states of high-density amorphous ice (HDA). Specifically, we study the historically most studied form as prepared following the protocol of pressure-induced amorphization of ice *I*_h_ at 77 K [30]. This so-called unannealed HDA (uHDA) is compared with expanded forms of HDA (eHDA), namely two different types of eHDA – one that is decompressed to 0.20 GPa and one that is decompressed to 0.07 GPa at 140 K. These sub-states of HDA are also compared with very-high density amorphous ice (VHDA), which does not belong to the HDA family of states [6].

For ice phases, the phase transition temperatures (e.g. melting or polymorphic transitions) typically shift by about 3–4 K from H_2_O to D_2_O ice [39]. For the eHDA samples we have taken this effect into account by preparing D_2_O samples at 143 K, HDO samples at 141.5 K and H_2_O samples at 140 K. The technique of isotope substitution neutron diffraction relies on a single structural model fitting to data obtained from H_2_O, D_2_O and HDO simultaneously. This may be jeopardised by strong isotope effects and quantum effects at cryogenic conditions. For liquid water the isotope effect on the structure is typically small [40]. At 77 K the dynamics show a significant isotope enhancement by 2 orders of magnitude and more [41], but the static structure is still barely affected. This holds true both for ice phases such as hexagonal ice that show anomalous negative thermal expansion at 77 K [42] and others that show regular thermal expansivity, e.g. high-pressure ice phases [39]. This anomalous behaviour is unrelated to quantum effects, but in fact a property of the hydrogen-bond network. In case of ice *I*_h_ the kinetically hindered H-ordering transition to thermodynamically stable ice XI, i.e. H-atom frustration, is at the origin of this anomaly [43]. In case of amorphous ices such H-atom frustration at 77 K was recently ruled out [24]. Consequently, we find in our work that one structural model fits for all isotopologues of eHDA and uHDA within small errors. This is in full agreement with earlier work on the topic of isotope substitution neutron diffraction of amorphous ices [44], including our own work [5,7,8]. The structural differences between the several variants of HDA studied in the present work, however, are clearly beyond the error of the structural model itself, see Figure 8. Further, the structural differences determined here by neutron scattering are consistent with those obtained from X-ray scattering [17].

Fully corrected interference differential scattering cross section data, *F*(*Q*), as well as site-site radial distribution function *g*(*r*) demonstrate that eHDA(0.20 GPa) neither clearly belongs to the HDA nor the VHDA family of states. In fact, it is roughly halfway between the two as seen, e.g. in *g*(*r*). In agreement with this conclusion, the transformation temperature for eHDA(0.20) of 131 K is in between the ones for VHDA of 126 K and fully relaxed eHDA of 134 K [20]. By contrast, eHDA(0.07 GPa) belongs to the HDA family as evidenced by the presence of a single interstitial water molecule [5]. Consequently, it is of utmost importance to decompress to beyond 0.20 GPa to make well-relaxed eHDA. Decompression of eHDA to 0.07 GPa is best, but comes with the risk of the immediate vicinity of the HDA-LDA transformation. Tonauer *et al.* have shown that nanosized domains of LDA start to form upon decompression just below 0.20 GPa and have an impact on the crystallization line [22]. If there are pressure and/or temperature gradients in the sample it is possible that parts of the sample already start to convert to LDA, so that the sample might end up as a mixture of eHDA and LDA. To play it safe we recommend decompression to 0.10 GPa. At this pressure the conversion to eHDA is already at completion [20], but the polyamorphic transformation to LDA is sufficiently far away.

Well-relaxed eHDA, prepared by decompression to 0.07 GPa here, shows some structural differences to uHDA at short and intermediate length scale. Comparing the two derived *g*_oo_(*r*) distributions, eHDA shows an enhancement around 3.6 Å, while uHDA a stronger contribution at values between 4 and 5.2 Å, indicating that uHDA contains an increased amount of tetrahedrality, which is in good agreement with recent X-ray scattering results [17]. Comparing the coordination numbers of the two HDA states, barely any difference is visible up to *r* = 5 Å – which also comprises the interstitial positions near 4 Å. This is evidence for eHDA(0.07 GPa) and uHDA to both belong to the HDA family. Beyond *r* = 6 Å the coordination number between the two differs distinctly. The reason for the much increased thermal stability of eHDA is found at a possibly increased amount of tetrahedral motives within the uHDA structure and on intermediate length scales beyond 6 Å. The relaxed nature of the H-bonded network at these length scales is the source for the high thermal stability that allows it to be heated into the ultraviscous liquid domain without occurrence of the polyamorphic transition to LDA.

## Disclosure statement

No potential conflict of interest was reported by the authors.
